# Supplementary material for: Proteomic identification of Axc, a novel beta-lactamase with carbapenemase activity in a meropenem-resistant clinical isolate of Achromobacter xylosoxidans
Source: Sci Rep. 2018 May 25;8:8181. doi: 10.1038/s41598-018-26079-z (PMC5970244; doi:10.1038/s41598-018-26079-z)
Supplement: Supplementary file 1 — Supplemental information [file 41598_2018_26079_MOESM1_ESM.pdf]

Supplemental material

Proteomic identification of Axc, a novel beta-lactamase with carbapenemase activity in a meropenem-resistant clinical isolate of *Achromobacter xylosoxidans*

Frank Fleurbaaij, Alex A. Henneman, Jeroen Corver, Cornelis W. Knetsch, Wiep Klaas Smits, Sjoerd T. Nauta, Martin Giera, Irina Dragan, Nitin Kumar, Trevor D. Lawley, Aswin Verhoeven, Hans C. van Leeuwen, Ed J. Kuijper and Paul J. Hensbergen

Supplemental Table 1 : Antibiograms obtained with Vitek 2 for the two clinical isolates of *Achromobacter xylosoxidans.* Eucast (http://www.eucast.org/) does not provide recommendations for clinical breakpoints and epidemiological cut-off values (ECOFF) for *A. xylosoxidans,* except for colistin. Using clinical breakpoints for other non-fermentative Gram-negative rods, R (resistant), S (sensitive), and I (intermediate) were scored.

	**First isolate**		**Second isolate**	
	Vitek value	Interpretation	Vitek value	Interpretation
Ampicillin	>=32	R	>=32	R
Augmentin	8	I	>=32	R
Colistin	2	S	<= 0.5	S
Ciprofloxacin	>=4	R	>=4	R
Cefepime	>=64	R	>=64	R
Cotrimoxazol	<=20	S	<=20	S
Cefotaxime	>=64	R	>=64	R
Cefuroxime	>=64	R	>=64	R
Ceftazidime	16	R	16	R
Cefoxitin	>=64	R	>=64	R
Gentamicin	>=16	R	>=16	R
Imipenem	8	R	8	R
**Meropenem**	**0.5**	**S**	**32**	**R**
Nitrofurantoin	256	R	256	R
Norfloxacin	>=16	R	>=16	R
Piperacillin/Tazobactam	<=4	S	8	I
Cefuroxim	>=64	R	>=64	R
Tobramycin	>=16	R	>=16	R
Trimethoprim	>=16	R	>=16	R

Supplemental figure S1: Quantitative proteomic analysis of the meropenem susceptible and resistant *Achromobacter xylosoxidans* clinical isolates.

A: Whole cell lysates from the two isolates (AchroS and AchroR, Table 1) were analyzed by SDS-PAGE and visualized by Coomassie staining. See supplemental figure S4 for a full gel picture, including MW markers.

B:Lanes from panel A were sliced into small bands and proteins were subsequently digested using trypsin. Following LC-MS/MS analyses and protein identification, spectral count analysis was used for relative protein quantification. Each dot represents the number of spectral counts observed per unique protein. Axc is the protein with the biggest difference in protein abundance between the resistant and susceptible isolate. In Table S2 and figure S2, the spectral counts and Qntdiff output can be found, respectively.

Supplemental figure S2: Analysis of differential proteins based on spectral counting using Qntdiff

The scatterplots display all quantified proteins using each protein's AchroS spectral count as x-coordinate and the corresponding ArchroR spectral count as y-coordinate, revealing a linear nature in the correlation between the spectral count values for each protein in both isolates. We devised a simple differential detection model that lumps all technical and biological variation into a null distribution for the perpendicular distances of proteins to a linear regression line through the data. If samples are similar enough, this method results in a p-value for the deviation from linearity of each protein. This method is implemented in our program Qntdiff. In figures A and B this method is applied to the in-gel digested data-set and shows differential proteins for p-value thresholds of 0.05 and 0.01, respectively. Figures C and D show the differential proteins selection in the in-solution digestion data-set for p-value thresholds of 0.05 and 0.01, respectively.

Supplemental figure S3: Presence of *axc* and sequence of its regulatory region in the meropenem resistant and susceptible clinical isolates

A: PCR using primers AxFor and AxRev (Table 3) showed that *axc* is present in both the meropenem resistant (AchroR) and meropenem susceptible (AchroS) clinical isolate. See Figure S4 for a full gel picture from which the cropped version shown is derived.

B: Sequence analysis showed that the intergenic region between *axc* and the gene encoding its putative repressor (*axcR*), were identical in AchroS and AchroR. At two positions (in red) this sequence differed from *A. xylosoxidans* strain NH44784-1996).

Supplemental figure S4: Full size gel pictures belonging to Fig. S1A and S3A

A

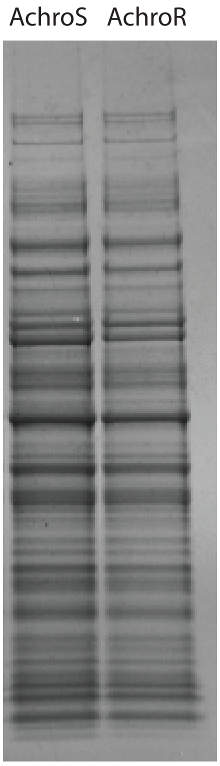

B

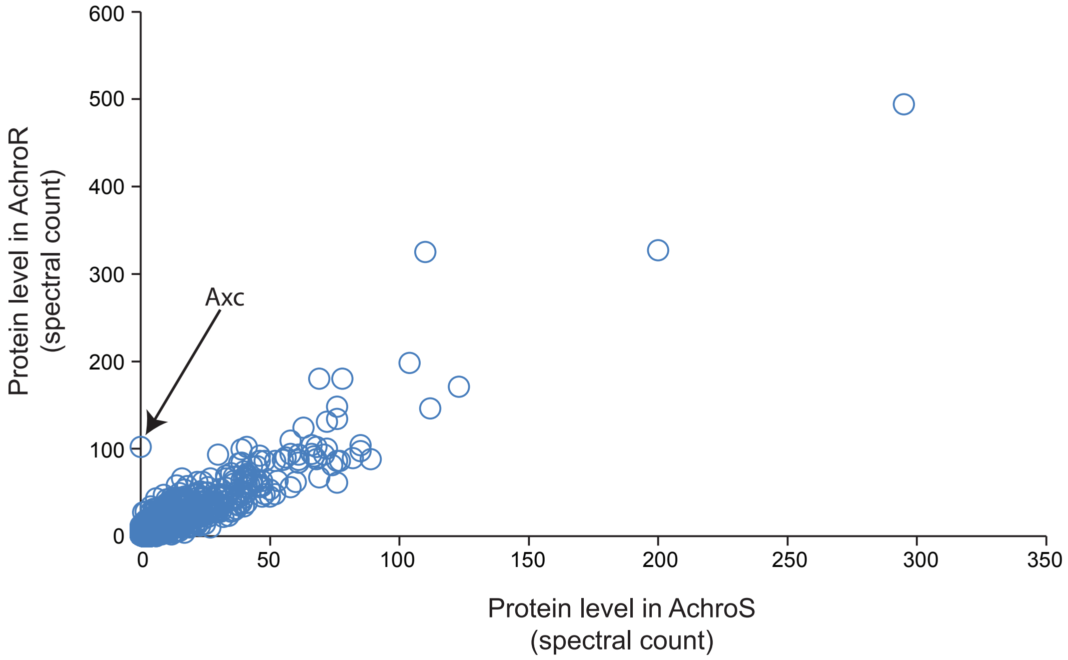

Supplemental figure S1

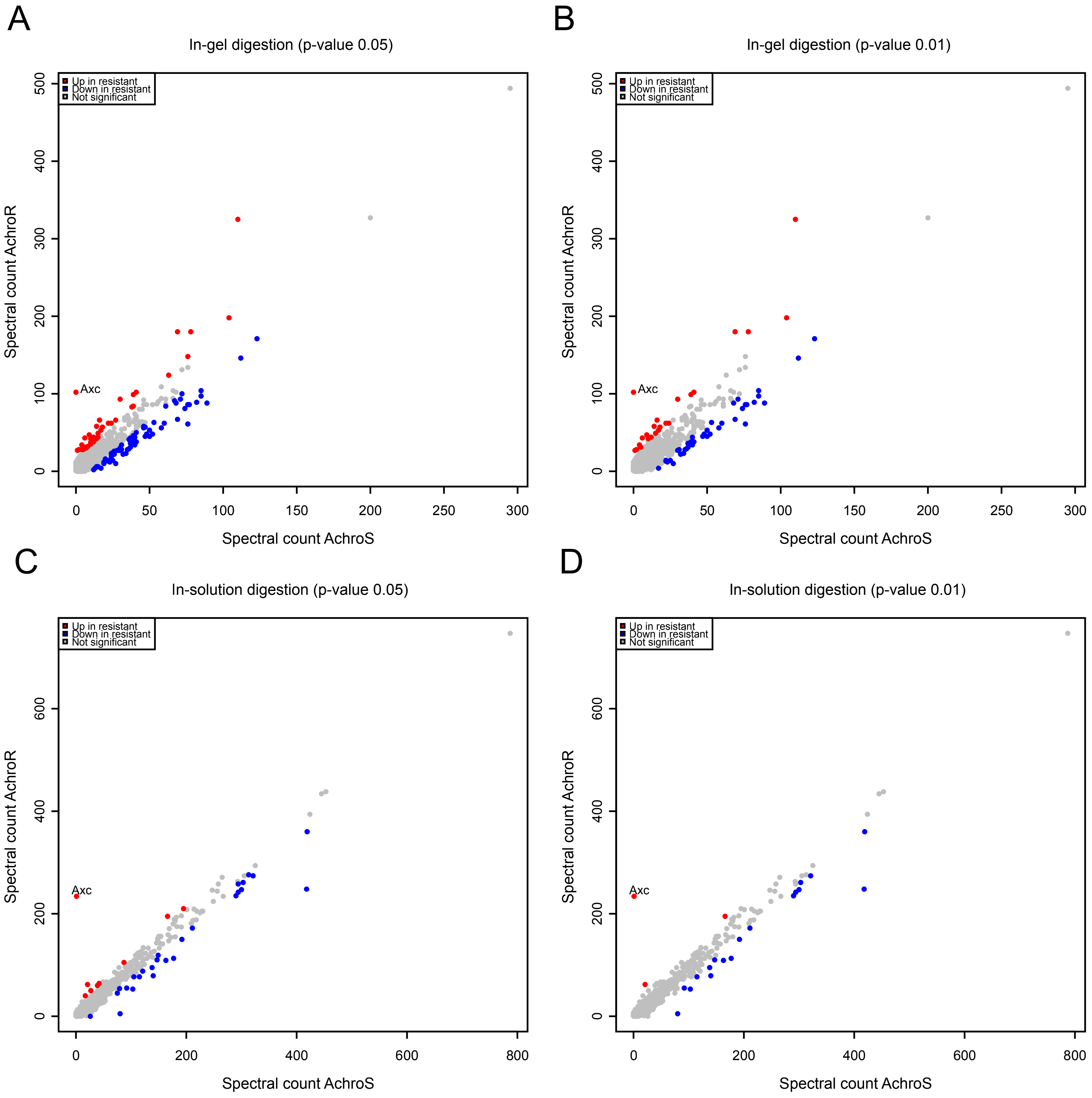

Supplemental figure S2

A

Axc PCR

AchroS AchroR

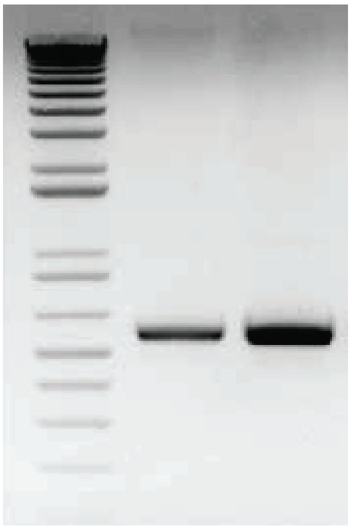

B

Intergenic region

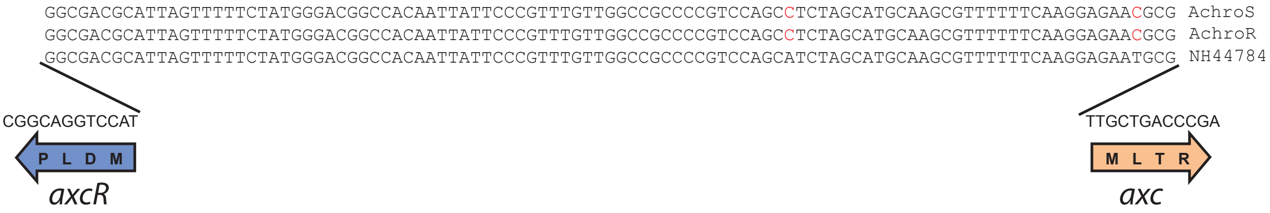

Supplemental figure S3

Full size gel pictures belonging to fig. S1A and S3A

Figure S1A

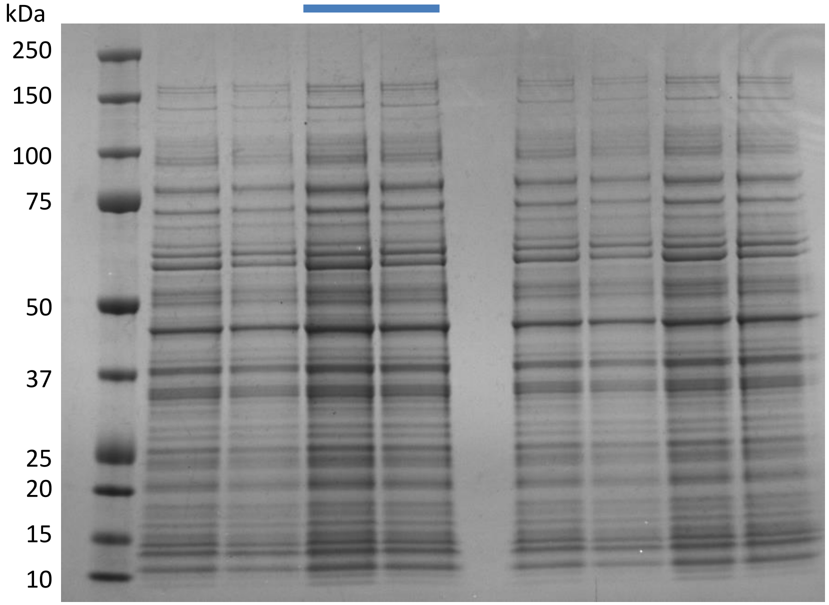

Figure S2A

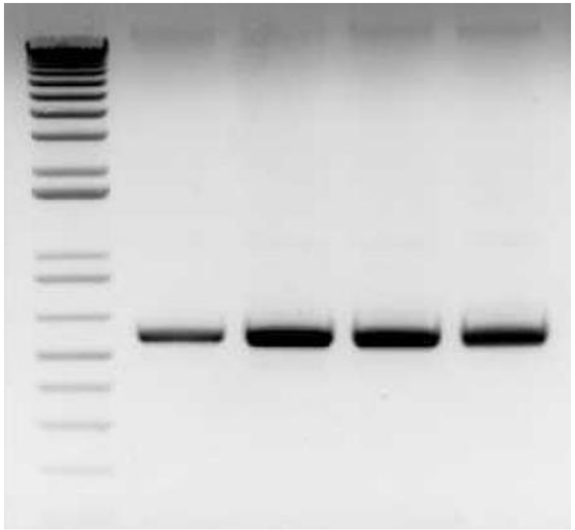

Supplemental figure S4
